# Improving smoking cessation after myocardial infarction by systematically implementing evidence-based treatment methods

**DOI:** 10.1038/s41598-021-04634-5

**Published:** 2022-01-12

**Authors:** Margret Leosdottir, Sanne Wärjerstam, Halldora Ögmundsdottir Michelsen, Mona Schlyter, Emma Hag, John Wallert, Matz Larsson

**Affiliations:** 1 Department of Clinical Sciences Malmö, Lund University, Malmö, Sweden; 2 Department of Cardiology, Skane University Hospital, Jan Waldenströms gata 15 plan 3, 205 02 Malmö, Sweden; 3 Department of Internal Medicine, Helsingborg Hospital, Helsingborg, Sweden; 4 Department of Internal Medicine, County Hospital Ryhov, Jönköping, Sweden; 5 Department of Clinical Neuroscience, Centre for Psychiatry Research, Karolinska Institutet, Solna, Sweden; 6 Clinical Health Promotion Centre, Lund University, Lund, Sweden; 7 The Heart, Lung and Physiology Clinic, Örebro University Hospital, Örebro, Sweden

**Keywords:** Cardiology, Risk factors, Acute coronary syndromes, Medical research

## Abstract

We compared the odds of smoking cessation at 2-months post-myocardial infarction (MI), before and after implementing routines optimizing use of evidence-based smoking cessation methods, with start during admission. The following routines were implemented at six Swedish hospitals: cardiac rehabilitation nurses offering smokers consultation during admission, optimizing nicotine replacement therapy and varenicline prescription, and contacting patients by telephone during the 1st week post-discharge. Using logistic regression, odds for smoking cessation at 2-months before (n smokers/n admitted = 188/601) and after (n = 195/632) routine implementation were compared. Secondary outcomes included adherence to implemented routines and assessing the prognostic value of each routine on smoking cessation. After implementation, a larger proportion of smokers (65% vs. 54%) were abstinent at 2-months (OR 1.60 [1.04–2.48]). Including only those counselled during admission (n = 98), 74% were abstinent (2.50 [1.42–4.41]). After implementation, patients were more often counselled during admission (50% vs. 6%, *p* < 0.001), prescribed varenicline (23% vs. 7%, *p* < 0.001), and contacted by telephone post-discharge (18% vs. 2%, *p* < 0.001). Being contacted by telephone post-discharge (adjusted OR 2.74 [1.02–7.35]) and prescribed varenicline (adjusted OR 0.39 [0.19–0.83]) predicted smoking cessation at 2-months. In conclusion, readily available methods for aiding smoking cessation can be implemented effectively in routine practice, with beneficial effects for post-MI patients.

## Introduction

Smoking is one of the primary risk factors for coronary heart disease (CHD)^1^. For smokers who suffer a myocardial infarction (MI), ceasing to smoke after the MI is the most effective of all preventive measures. Similarly, tobacco abstinence is associated with a lowered risk of reinfarction by 30–40%^2,3^ and death by 45%^4^. Supporting patients to quit smoking after an MI, and to remain abstinent long-term, are therefore primary goals of cardiac rehabilitation (CR).

The Swedish quality registry SWEDEHEART (The Swedish Web-system for Enhancement and Development of Evidence-based care in Heart disease Evaluated According to Recommended Therapies) registers data from an unselected MI-patient population, with nationwide coverage and one-year follow-up data available for > 80% of those < 80 years of age (www.swedeheart.se). According to the most recent SWEDEHEART annual report, the proportion of active smokers among patients with MI in Sweden was 23%, a proportion which has decreased successively over the last decade—alongside a general population reduction in daily smoking^5,6^. The proportion of smokers who reported being abstinent at 2-months and 1-year post-MI was 57% and 56%, respectively, which is somewhat higher than the European average of ~ 45%^6,7^. However, despite the decrease in active smokers at baseline, no progress in smoking abstinence rates among Swedish patients with MI has been observed for more than ten years^6^.

Current CR guidelines advocate professional support, with a smoking cessation programme initiated already at hospital admission^8,9^. Drug intervention is also strongly recommended, using nicotine replacement therapy (NRT), varenicline and/or bupropion^8,9^. Varenicline is the most effective medical treatment to support smoking cessation^10^. Recent studies have confirmed its safety not only in patients with stable CHD but also in patients with acute coronary syndromes, including MI^11–13^. In the fifth and most recent European Action on Secondary and Primary Prevention by Intervention to Reduce Events (EUROASPIRE) survey, a majority of currently smoking patients with MI were offered personal advice by a healthcare professional to stop smoking but NRT was prescribed to only 7% of smokers and varenicline to 2%, with no meaningful change between EUROASPIRE III and V^7,14^. Consequently, there is clearly a considerable room for improvement with respect to medical treatment for active smokers with MI.

The primary aim of this study was to compare the odds of smoking cessation at 2-months post-MI before versus after implementing a set of pre-specified routines for optimization of evidence-based treatment methods for smoking cessation, with start during admission (1) for all currently smoking patients admitted before versus after implementation and (2) for all currently smoking patients admitted before implementation versus a subgroup of patients admitted during the implementation period who received short smoking cessation counselling during admission. The secondary aims were to assess (3) adherence to implemented routines and (4) the prognostic value of each routine on smoking cessation.

## Methods

### Study design

A retrospective observational multicentre cohort study.

### Setting and participants

As a joint venture for clinical quality improvement, structured routines for early smoking cessation counselling and treatment optimization were implemented at six hospitals in Sweden in 2018–2019. Two are university hospitals. The largest hospital is situated in Malmö, the third largest city in Sweden. Two are of medium size (Örebro and Jönköping) and three are smaller urban or rural hospitals (Trelleborg, Eksjö and Värnamo). The three largest hospitals have acute percutaneous coronary intervention facility, and all have a coronary intensive care unit and an outpatient cardiac rehabilitation (CR) centre. The CR team in Malmö was the first to start implementing the new working routines. The Malmö team thereafter visited the other hospitals to introduce the CR and coronary intensive care personnel to the new routines and support implementation.

The participating CR centres report to the SWEDEHEART registry^6^ and provide comprehensive CR to post-MI patients. The “usual care” CR programme starts with an individual patient assessment with a nurse at 2–3 weeks after discharge, followed by two registry-based nurse visits at 6–10 weeks (2-month visit) and 11–13 months (1-year visit). Central components of the programme include control and treatment of cardiovascular risk factors, interactive patient education, psychosocial management, smoking cessation counselling (individual or group therapy) if needed, individual patient assessment with a physiotherapist, and supervised centre-based exercise training.

### The new routines

The newly adapted routines included the following:

- A CR nurse should identify all actively smoking MI-patients while still hospitalized for their MI and offer a short consultation with the patient during admission. A new information pamphlet for patients was designed, which included information on the benefits of smoking cessation, tips for avoiding relapse after discharge, plan for follow-up, and contact information for the CR centre and generally available anti-smoking aid (telephone and online support).
- The CR nurse should give advice to the coronary intensive care nurses and physicians on optimal dosage of NRT during admission and encourage prescription at discharge (even though NRT is available over the counter in Sweden), to further promote adherence.
- The CR nurse should identify patients eligible for treatment with varenicline, and alert the responsible physician, who in turn initiates treatment if deemed suitable.
- Varenicline treatment should preferably start before discharge.
- The patient should be contacted by telephone, by the same nurse that consulted the patient during admission, during the first week after discharge. Thereafter, usual care CR commenced.
- If the patient opted out from varenicline treatment during admission and was still smoking at the telephone contact or 1st CR follow-up visits, treatment should be offered again.
- Finally, centres were encouraged to strive for continuity in nurse-patient care, meaning the same CR nurse should care for the patient from the first contact during admission throughout CR follow-up.

Consequently, the CR nurse was the key professional in implementing the new routines. CR nurses at all participating centres had tobacco counselling qualifications prior to project initiation. If requested, a brief refreshment course and coaching guide was provided by a tobacco counsellor. At some centres, the routines were predominantly new, and at others only smaller amendments or refreshing of routines were needed (see Supplementary Table S1). Whether patients qualified for varenicline treatment and which treatment was offered (NRT, varenicline, or both) was decided by the treating physician. The type of NRT used at each hospital followed local subvention agreements and was not centrally regulated.

### Data collection and outcomes

Smoking cessation at 2-months post-MI for currently smoking patients aged 18–74 years admitted to the coronary intensive care units at the participating hospitals with a type-1 MI, before (reference period) and after (implementation period) routine implementation, were compared. The implementation and reference periods were of equivalent length at each participating hospital. For the three hospitals with the longest implementation periods (8–14 months), the end of the reference period was set one month prior to start of the implementation period. The remaining three hospitals had shorter implementation periods (4 months), and for which the same period the year prior to the implementation year, was used to minimize seasonal bias. The start and length of the study periods per hospital is listed in Supplementary Table S2. Baseline data and smoking status at 2-months was retrieved from the SWEDEHEART registry. Current smoking at baseline and follow-up was self-reported and defined as daily smoking during the last month. Active smokers at baseline were identified by the admitting doctor and/or coronary intensive care unit nurse according to local routine at each hospital. Adherence to the pre-set routines was retrieved from patient journal records at each participating hospital.

According to the original study protocol, primary outcome was smoking abstinence at the 1-year follow-up visit. Since the Covid-19 pandemic started at the time the last patients included in the implementation period had their 2-month follow-up—seriously debilitating planned follow-up routines at all CR centres—primary outcome was changed to smoking abstinence at 2-months post-MI. Secondary outcomes included (1) adherence to the newly implemented routines and (2) prognostic value of each of the new routines for smoking abstinence at 2-months. Continuity in nurse-patient care was defined as 2/3 or more of same nurse-patient contact (physical visits during admission and follow-up and telephone contact during follow-up).

### Ethics approval and consent to participate

The Swedish Ethical Review Authority approved the study (2019–06177). The study was carried out in accordance with relevant guidelines and regulations. The need for signed consent by patients for inclusion in Swedish quality registries has collectively been waived in Sweden (The Patient Data Legislation 2008:355). Instead, a nurse or physician verbally informs all eligible patients at hospital admission of their registration in SWEDEHEART and opt-out rights. All patients have the right to deny registration and retain the right to have their data removed from the registry at any time.

### Sample size calculations

In the randomized controlled double-blind Evaluation of Varenicline in Smoking Cessation for Patients Post-Acute Coronary Syndrome (EVITA) trial, patients with acute coronary syndrome and who were smokers at baseline were randomized to receive either varenicline or placebo before discharge^11^. In the intervention arm, 58% of patients were smoke-free at first follow-up (12 weeks), compared to 36% in the placebo arm (21.3% difference, *p* < 0.0001). In the current study, at the CR centres implementing the new routines, 59% of current smokers at the time of MI were abstinent at the first SWEDEHEART follow-up (2 months) in 2017^15^. Based on EVITA results, we concluded that a 15%-point change improvement in smoking cessation rates was a reasonable target for the current study. A lower improvement rate compared to EVITA was expected because the proportion of smokers abstinent at 2-months was already high and not all patients were expected to be identified and thus potentially benefitting from the new routines. With a power of 80% and a two-sided significance level of 0.05 we calculated that we needed to include 150 patients in each group^16^.

### Statistical analysis

Baseline patient characteristics are presented as medians (q1, q3) and percentages. Adherence to the implemented routines is described using percentages and comparisons made using the chi-square test. The primary and secondary outcomes were analyzed using logistic regression. The primary outcome analysis was done in two steps: comparing all current smokers admitted during the reference period to (1) all current smokers admitted during implementation period, and (2) current smokers that were subject to counselling during admission during the implementation period only. For the secondary outcomes, each routine´s association with smoking cessation at 2-months was estimated (1) on data from the implementation period alone, and (2) including all patients from both periods. For all regression analyses, cases with missing data were excluded. Crude models were fitted, whereafter models were adjusted for age, gender, size of center (small, medium, large), length of hospital stay, and whether the patient was discharged during the weekend (Saturday-Sunday), Mondays or during holidays. Mondays were included as patients discharged on Mondays often had returned home before the nurse had a chance to consult the patients.

## Results

**Figure 1:**
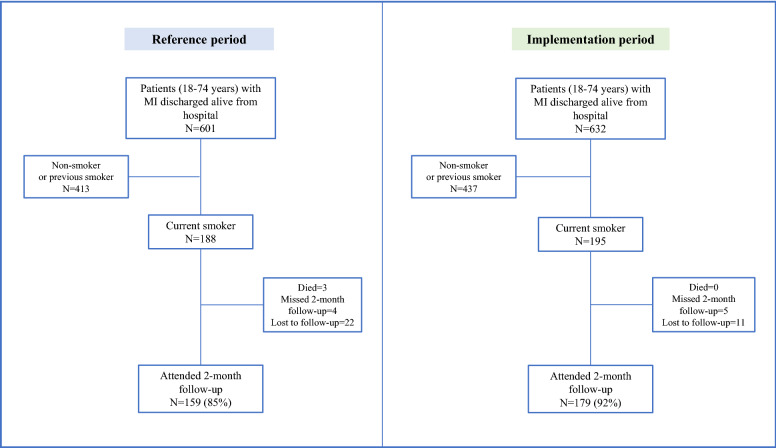
Flow chart for patients in the study. MI, myocardial infarction.

Implementation of the new routines started in Malmö in October 2018 with the last hospitals (Eksjö and Värnamo) joining in September 2019 (see Supplementary Table S2). The time frame for patient inclusion ended early January 2020, when 195 current smokers had been admitted for MI (Fig. 1). During the pre-specified reference period, 188 active smokers were admitted. A slightly higher number of patients were included in the analysis than indicated by the sample size calculations, to compensate for attrition. Baseline characteristics are shown in Table 1. Median age was 60 (range 33–74) years in both groups. The proportion of men was 77% in the reference group and 71% in the intervention group (*p* = 0.1). Most follow-up visits were conducted in-person (98% during the reference period and 99% during the implementation period, *p* = 0.2). In-person visits took place at the hospital.

**Table 1:** Baseline characteristics for smokers discharged from participating hospitals during the reference and implementation periods.

	Reference period	Implementation period
Number	188	195
Women (%)	23	29
Age (years)	60 (53, 67)	60 (53, 66)
Weight (kg)	83.5 (73.0, 92.0)	82.0 (70.0, 90.8)
BMI (kg/m2)	27.6 (24.1, 30.1)	27.1 (24.3, 29.9)
Using wet snuff (%)	7	9
Prior disease history		
Diabetes (%)	22	21
Hypertension (%)	43	42
CHD (%)	18	20
STEMI (%)	51	56
Max TnT (ng/L)	849 (154, 3505)	1305 (294, 4320)
Reduced LVEF (< 50%) (%)	30	27
Number of days in hospital	4 (3, 5)	4 (3, 6)
Discharged during weekends, Mondays, or holidays (%)	23	24

Numbers represent percentages (%) and median (q1, q3) values.

*Kg* kilograms, *BMI* body mass index, *m2* square meters, *CHD* coronary heart disease, *STEMI* ST-elevation myocardial infarction, *TnT* troponin T, *LVEF* left ventricular ejection fraction.

### Primary outcome

**Figure 2:**
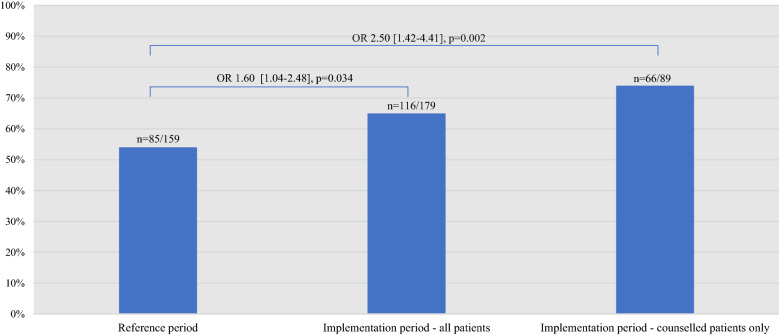
The proportion of abstinent patients and the crude odds of smoking abstinence at 2-month follow-up during the reference period (far left) and implementation period, for all patients (center) and for those counselled during admission only (far right). OR, odds ratio.

In total, 159 out of the 188 (85%) and 179 out of the 195 (92%) active smokers at baseline during the reference and implementation periods, respectively, attended a 2-month follow-up (338 patients in total). All of these had outcome variable data, while 5 patients (1.5%) had missing data on covariates, leaving 333 patients in the final sample. Significantly more patients were abstinent from smoking at the 2-month follow-up after the new routines had been implemented: 64% compared to 54% during the reference period (10-percentage point difference, crude OR 1.60 [1.04–2.48], *p* = 0.034; adjusted OR 1.60 [1.03–2.52], *p* = 0.037;) (Fig. 2). Including implementation period patients who were counselled by a CR nurse during admission only (n = 89), 73% were abstinent at the 2-month follow-up, compared to 54% out of all patients included during the reference period (19-percentage point difference, crude OR 2.50 [1.42–4.41], *p* = 0.002; adjusted OR 2.68 [1.46–4.91], *p* = 0.002).

### Secondary outcomes

When assessing adherence to the implemented routines all currently smoking patients during both periods were included (n = 188 and n = 195). After implementation patients were more often counselled during admission (50% (n = 98) vs. 6% (n = 11), *p* < 0.001), and prescribed varenicline at discharge or during follow-up (23% (n = vs. 7% (n = 12), *p* < 0.001) compared to before. There was no difference in the proportion of patients receiving an NRT prescription at discharge or during follow-up (23% (n = 45) vs. 16% (n = 30), *p* = 0.093). During the implementation period, patients were more often contacted by telephone during the first post discharge week, compared to the reference period (18% (n = 35) vs. 2% (n = 4), *p* < 0.001). The total number of contacts (by telephone or visits) during the 2-month follow-up period was higher during the implementation period (2.8 vs. 2.3 contacts, *p* < 0.001) and more patients had continuity in their nurse-patient care (64% (n = vs. 51%, *p* = 0.010). Comparing patients counselled during admission versus those not counselled, 33% (n = 32) versus 14% (n = 13) received a varenicline prescription at discharge or during follow-up (*p* = 0.002), 30% (n = 29) versus 17% (n = 16) received a NRT prescription at discharge or during follow-up (*p* = 0.041), and 36% (n = 35) versus 0% (n = 0) were contacted by telephone during the first week after discharge (*p* < 0.001). Patients not counselled during admission more often had short hospital stays (≤ 3 days, 51% (n = 48) versus 32% (n = 31), *p* = 0.001) and were more often discharged during weekends/Mondays/holidays (33% (n = 32) versus 15% (n = 15), *p* = 0.004). Three patients refused counselling.

Results from secondary regression analyses are shown in Table 2 and Supplementary Table S3. In crude models using data from the implementation period only, being (1) counselled during admission, (2) contacted during the 1st week after discharge, and (3) having nurse-patient care continuity each positively predicted 2-month smoking cessation, while (4) being prescribed varenicline at discharge or during follow-up negatively predicted 2-month smoking cessation. Adjusting for covariates, contact during the 1st week after discharge and prescription of varenicline remained statistically significant. In crude analyses of the whole cohort (reference and implementation periods combined) being (1) counselled during admission, (2) contacted during the 1st week after discharge, and (3) having nurse-patient care continuity positively predicted 2-month smoking cessation, while being prescribed NRT at discharge or during follow-up negatively predicted 2-month smoking cessation (Supplementary Table S3), all remaining significant in the adjusted model.

**Table 2:** Odds ratios for smoking abstinence at 2-months post-MI for patients exposed to each implemented routine.

Implemented routine	n = 179 (yes/no/missing)	Crude		Adjusted*	
		OR [95% CI]	*p*	OR (95% CI)	*p*
Counselling during hospital admission	89/90/0	2.30 [1.22–4.31]	0.010	1.87 [0.94–3.74]	0.076
Contact during the 1st week post-discharge	32/145/2	2.73 [1.06–7.04]	0.038	2.74 [1.02–7.35]	0.046
Prescription of varenicline at discharge or during follow-up	42/136/1	0.39 [0.19–0.80]	0.010	0.39 [0.19–0.83]	0.015
Prescription of NRT at discharge or during follow-up	42/136/1	0.75 [0.37–1.53]	0.431	0.64 [0.29–1.41]	0.269
Continuity in nurse-patient care**	119/60/0	2.10 [1.11–3.99]	0.024	1.88 [0.94–3.76]	0.073

Crude and adjusted odds ratios are shown. Only patients from the implementation period are included, comparing those exposed to those not exposed.

*OR* odds ratio, *CI* confidence interval, *NRT* nicotine replacement therapy.

*Adjusted for age (year), gender (male/female), size of hospital (small, medium, large), length of hospital stay (days), and whether the patient was discharged during the weekend or during holidays (yes/no).

**At least two-thirds of nurse-patient contacts (physical visits/telephone) with same nurse.

Being a retrospective study, no harms, or unintended effects on account of the current study were noted or documented.

## Discussion

The present study showed that implementing evidence-based, low cost- and readily available methods for smoking cessation can seemingly have a substantial impact on smoking habits after an MI. Implementation resulted in a higher proportion of abstinent patients at 2-month follow up; 64% versus 54% during the reference period, and including only patients counselled during admission smoking abstinence this difference was 73% versus 54%.

To stop smoking following onset of CHD or after undergoing revascularization sharply reduces the risk of reinfarction and death. Information and support for smoking cessation should be delivered routinely to all patients with a diagnosis of CHD^3,9^. This risk reduction is evident across strata by sex, age, index cardiac event, country, and year of study initiation^3^.

According to SWEDEHEART data, being abstinent from smoking at 2-months after an MI is associated with a 50% reduction in 10-year mortality risk^6^. The population of patients as well as the structure of follow-up in SWEDEHEART is practically equivalent to the present study´s control period population. Hence, more abstinent patients 2-months after MI should lead to a significant improvement in long-term life expectancy.

At all six hospitals participating in the project, various forms of smoking cessation support were already in-place before project initiation. Thus, the implemented model was largely an implementation that strengthened and structured existing support. Indeed, when analyzing data from both periods jointly the prognostic value of each individual routine was generally stronger than for the implementation period alone. Meanwhile, the smoking cessation rate during the implementation period, when the routines were applied in a more systematic way, was significantly higher. Focus during the implementation period was on assessing smoking status early, systematically offering all current smokers brief verbal advice complemented with written material and optimizing pharmacological treatment. Implementation was performed without increasing personnel resources—and although it is difficult to estimate the exact cost for society—there is little doubt that a nurse-managed smoking cessation program after acute MI is cost-effective. In a study by Quist-Paulsen and colleagues, the cost of a nurse-led smoking intervention program compared favourably to other treatment modalities for CHD patients, being approximately 1/25 the cost of statins and angiotensin-converting enzyme inhibitors^17^. In another study by Krumholz et al. that evaluated the cost-effectiveness of a similar nurse-led intervention, which included three hours of extra counselling time with a nurse, was cost-effective at an estimated $220/year of life saved^18^. In the present study we estimate that the extra counselling time (patient identification, counselling during admission and more telephone contacts during follow-up) to be approximately 1–2 h per patient. More time and resources for counselling in future studies and clinical praxis is most likely warranted, also since clinical studies show a positive relationship between number of counselling sessions and abstinence rate^19,20^. To achieve optimal effect, smoking cessation programmes should preferably include wide-ranging medical information, behavioral aspects, community-oriented methods, and strengthened pharmaco-therapeutic treatments^8,19,21^. Moreover, it has been proposed that telephone counselling and text messaging is effective for outpatients after a MI^22^.

Out of all current smokers at baseline less than 10% were concomitantly using wet snuff during the reference and implementation periods, respectively. Use of wet snuff is common in Sweden, with 18% of men and 5% of women using wet snuff daily^5^ and using wet snuff as means to quit smoking cigarettes is quite common. The small number of current wet snuff users in our study, however, limited the possibilities for meaningful sub-analyses of this group.

As the CR nurses at the participating hospitals only work office hours Monday to Friday, lack of time to identify and subsequently counsel patients during admission was probably the major barrier to offering counselling during admission to more patients, but only 50% of the patients included in the current analysis received counselling. This in turn affected the CR nurses´ possibility to improve treatment during admission and at discharge. Also, if the patients were not identified during admission, they were not contacted by telephone during the first week after discharge. Instead, first contact was at the initial CR assessment visit (2–3 weeks post discharge), as it was during the reference period and which is the general rule in Sweden. The importance of identifying patients early is supported in our data by the large difference in the odds for smoking cessation when only including patients who received counselling (OR 2.50 [1.42–4.41], *p* = 0.002) versus all patients treated during the implementation period (OR 1.60 [1.04–2.48], *p* = 0.034). Given the potential benefits in saved lives and cost-effectiveness of a relatively simple intervention, increasing CR nurse resources to facilitate counselling even on weekends and during odd hours should be a priority.

Sweden has a low prevalence of daily smokers (~ 7%) yet the proportion of MI patients that smoke is 23%^5,6^. Many European countries have significantly higher prevalence of smoking among MI patients, including Spain (41%), Turkey (42%), Serbia (46%) and Cyprus (57%)^23^. Accordingly, simple interventions such as the one evaluated in the current study, in countries with a high proportion of active smokers, could have major effects on the general prognosis post-MI. We encourage healthcare authorities to facilitate implementation of structured multi-component smoking cessation methods, which should be offered to every smoker who suffers an MI.

In our study, being prescribed varenicline and NRT negatively predicted smoking cessation at 2-months. This might seem controversial, as varenicline and NRT treatment are effective smoking cessation treatment modalities and come highly recommended^10,11,24^. The most plausible explanation for our findings is that varenicline and NRT were prescribed according to individual patient´s indications, and not offered to all patients in an unselected way. This leads to a selection bias, where patients with the strongest nicotine addiction and/or longest smoking history are offered treatment. The fact that the overall smoking cessation rates during the implementation period (when varenicline was considerably more often prescribed) was significantly higher than during the reference period, supports the use of medical therapy when indicated.

The study has several strengths and limitations. The study had a relatively large sample size and several hospitals, both university hospitals and rural hospitals, were involved. All currently smoking MI patients admitted at the participating hospitals were included, increasing study representativeness. Missing covariate data was also minimal. On the other hand, smoking status was assessed by self-report and no biochemical validation was performed to verify abstinence.. The duration and intensity of smoking (i.e., n packages smoke per time unit, and years of smoking) is not available via SWEDEHEART or medical records. Some of the implemented routines were already in place at the participating hospitals before start of the project, also during the reference period. This reduced the difference between implementation and control periods as can be seen in Supplementary Table S3. Even though attendance at the 2-month follow-up CR visit during both treatment periods was high (85% and 92%) there is a potential risk for selection bias. As missing values were likely to be missing not at random (nonignorable nonresponse), and the fact that missingness was in the outcome variable, we decided against multiple imputation. Also, the potential bias from this should be on the conservative side, attenuating the estimates since a stronger self-selection of the healthiest, most motivated patients likely took place in the reference group, which had higher attrition rates, compared to the implementation group. Although the association of each component of the new routines was assessed in the secondary analysis collinearity is plausible. Higher order effects are also plausible. A future large-scale study that specifically probes effect modification and individual component role relative to the outcome seems warranted. The short follow-up of 2 months is a limitation. A longer follow-up of 6 or 12 months would have been preferred, as abstinence at 12 months is a good predictor for long-term abstinence^25^. However, as previously stated the Covid-19 pandemic made a longer follow-up period unfeasible. Other factors than the implemented routines, such as public health interventions or general attitudes in society might have changed during the periods and influenced the results. However, major confounding effects of this sort seem unlikely since SWEDEHEART data has shown similar results regarding smoking cessation after MI for ten years prior to and during the study period^6^.

In conclusion, our results from a real-life clinical setting show that implementing evidence-based and readily available methods for smoking cessation can seemingly have a significant positive impact on smoking cessation post-MI. The result was substantial even though far from all patients were subject to all implemented routines. The most plausible barrier to implementing the new routines was lack of time for the CR nurses. Strengthening CR nurse resources for this important task should thus be a priority.

## Supplementary Information

Supplementary Information.

## Data Availability

The SWEDEHEART registry data that support the findings of this study are available from Uppsala Clinical Research Center (UCR) in Sweden, but restrictions apply to the availability of these data, which were used under license for the current study, and so are not publicly available. Third-party data usage is not allowed, irrespective of whether the data contain potentially identifying sensitive data or not. Instead, given ethical study approval from the Swedish Ethical Review Authority, access to SWEDEHEART data supporting the present findings can be applied for from UCR. Further information can be found on the UCR (www.ucr.uu.se/en/) and Swedish Ethical Review Authority (etikprovningsmyndigheten.se) websites. The study protocol has not been previously published but is available from the corresponding author on reasonable request.

## Abbreviations

BMI: Body mass index
CHD: Coronary heart disease
CI: Confidence interval
CR: Cardiac rehabilitation
EUROASPIRE: European Action on Secondary and Primary Prevention by Intervention to Reduce Events
EVITA: Evaluation of Varenicline in Smoking Cessation for Patients Post-Acute Coronary Syndrome
Kg: Kilograms
LVEF: Left ventricular ejection fraction
MI: Myocardial infarction
NRT: Nicotine replacement therapy
OR: Odds ratio
STEMI: ST-elevation myocardial infarction
SWEDEHEART: The Swedish Web-system for Enhancement and Development of Evidence-based care in Heart disease Evaluated According to Recommended Therapies
TnT: Troponin T
