# Supplementary material for: Improving smoking cessation after myocardial infarction by systematically implementing evidence-based treatment methods
Source: Sci Rep. 2022 Jan 12;12:642. doi: 10.1038/s41598-021-04634-5 (PMC8755785; doi:10.1038/s41598-021-04634-5)
Supplement: Supplementary file 1 — Supplementary Information. [file 41598_2021_4634_MOESM1_ESM.pdf]

Supplementary information file

Improving smoking cessation after myocardial infarction by systematically implementing evidence-based treatment methods

Author list: Margret Leosdottir, Sanne Wärjerstam, Halldora Ögmundsdottir Michelsen, Mona Schlyter, Emma Hag, John Wallert, Matz Larsson

Supplementary Table S1. Smoking cessation treatment and care at each participating hospital before implementation of the new routines.

	CR nurses counsel patients during admission	Patients are offered NRT during admission	Varenicline is initiated before discharge	Varenicline is prescribed during follow-up	Smoking patients are contacted by telephone during 1st week post discharge	We strive for continuity in patient-nurse contact throughout follow-up
Malmö	orange	green	orange	yellow	orange	green
Örebro	yellow	green	green	green	yellow	green
Trelleborg	orange	green	orange	orange	orange	orange
Jönköping	orange	green	orange	yellow	orange	green
Värnamo	orange	green	yellow	yellow	orange	green
Eksjö	yellow	green	orange	yellow	orange	light green

CR, cardiac rehabilitation; NRT, nicotine replacement therapy. Orange = New routine; yellow = partly new routine; green = already implemented.

Supplementary Table S2. Inclusion time frames and number of patients for the reference vs implementation periods at each participating hospital.

	Reference period				Implementation period			
Hospital	Start date	Stop date	Total number of MI patients	n (%) current smokers	Start date	Stop date	Total number of MI patients	n (%) current smokers
Malmö	22-07-17	22-09-18	311	105 (34)	22-10-18	22-12-19	327	128 (39)
Örebro	07-12-17	07-12-18	152	44 (29)	07-01-19	07-01-20	145	41 (28)
Trelleborg	01-06-18	01-03-19	46	17 (37)	01-04-19	01-01-20	63	16 (25)
Jönköping	23-08-18	23-12-18	46	10 (22)	23-08-19	23-12-19	53	2 (4)
Värnamo	01-09-18	31-12-18	23	2 (9)	01-09-19	31-12-19	26	5 (19)
Eksjö	01-09-18	31-12-18	23	10 (43)	01-09-19	31-12-19	18	3 (17)
All			601	188 (31)			632	195 (31)

MI, myocardial infarction.

Supplementary Table S3. Odds ratios for smoking abstinence at 2-months post-MI for patients exposed to each routine, with patients from both periods (reference and implementation) included.

		**Crude**		**Adjusted***	
Routine	n=338 (yes/no/missing)	OR [95% CI]	p	OR (95% CI)	p
Counselling during hospital admission	98/237/3	2.49 [1.48-4.19]	0.001	2.53 [1.39-4.63]	0.003
Contact during the 1st week post-discharge	36/296/6	3.71 [1.50-9.17]	0.005	3.60 [1.41-9.19]	0.007
Prescription of varenicline at discharge or during follow-up	54/280/2	0.62 [0.34-1.11]	0.108	0.59 [0.32-1.10]	0.098
Prescription of NRT at discharge or during follow-up	69/265/2	0.58 [0.34-0.99]	0.045	0.49 [0.28-0.87]	0.014
Continuity in nurse-patient care**	212/126/0	1.59 [1.02-2.49]	0.04	1.63 [1.02-2.61]	0.043

Crude and adjusted odds ratios are shown. Patients exposed are compared to those not exposed. OR, odds ratio; CI, confidence interval; NRT, nicotine replacement therapy. *Adjusted for age (year), gender (male/female), size of hospital (small, medium, large), length of hospital stay (days), whether the patient was discharged during the weekend or during holidays (yes/no), and period of study (reference/implementation. **At least two-thirds of nurse-patient contacts (physical visits/telephone) with same nurse.
